# Combined SSTR2-targeted Analogue with ^177^Lu-DOTATATE radionuclide and octreotide therapy for refractory meningioma: a case report

**DOI:** 10.3389/fonc.2026.1820428

**Published:** 2026-06-11

**Authors:** Carlen A. Yuen, Mahbod Jafarvand, David O. Kamson, Eleanor Chu, Jennifer E. Soun, William H. Yong, Frank P. Hsu, Hansen Bow, Michelle Zheng, Shyam M. Srinivas

**Affiliations:** 1 Department of Neurology, Division of Neuro-oncology, University of California, Irvine, Irvine, CA, United States; 2 Chao Family Comprehensive Cancer Center, University of California, Irvine, Irvine, CA, United States; 3 Department of Radiology, Division of Nuclear Medicine, University of California, Irvine, Irvine, CA, United States; 4 The Sidney Kimmel Comprehensive Cancer Center, Johns Hopkins University, Baltimore, MD, United States; 5 Department of Neurology, School of Medicine, Johns Hopkins University, Baltimore, MD, United States; 6 Department of Radiology, Division of Neuroradiology, University of California, Irvine, Irvine, CA, United States; 7 Department of Pathology, Division of Neuropathology, University of California, Irvine, Irvine, CA, United States; 8 Department of Neurosurgery, University of California, Irvine, Irvine, CA, United States; 9 UC Irvine Charlie Dunlop School of Biological Sciences, Irvine, Irvine, CA, United States

**Keywords:** 177Lu-DOTATATE, meningioma, octreotide, peptide receptor radionuclide therapy (PRRT), somatostatin receptor type 2 (SSTR2)

## Abstract

Meningiomas are the most common primary intracranial tumor in adults. Beyond surgery and radiation, no standard of care therapy exists. Meningiomas overexpress somatostatin receptor 2 (SSTR2), providing the rationale for somatostatin analogue-based therapies, including octreotide. The addition of everolimus, a mammalian target of rapamycin inhibitor, to octreotide marginally improves the 6-month progression-free survival (PFS) rate. For this reason, novel therapies have emerged for the treatment of refractory meningiomas, including somatostatin receptor targeted radionuclide therapy. However, preliminary results with refractory meningiomas treated with single agent ^177^Lu-DOTATATE, a β-emitting peptide receptor radionuclide therapy (PRRT), demonstrated outcomes comparable to those observed with combination octreotide and everolimus. In contrast, in neuroendocrine tumors (NETs), octreotide combined with PRRT has shown significant prolongation of PFS compared to high-dose octreotide alone. The final analysis of the NETTER-1 trial also yielded a trend towards an improvement in median overall survival, supporting the FDA approval of ^177^Lu-DOTATATE in 2018 for use in NETs. We present the first case of a refractory meningioma patient treated with combination PRRT and octreotide in a 66-year-old male who received ^177^Lu-DOTATATE 7.4 GBq (200 mCi) and intramuscular long-acting octreotide 40 mg every 8 weeks for four cycles followed by a single cycle of octreotide 40 mg monotherapy. Treatment was discontinued due to his unfortunate death from non-treatment related causes, occurring 2.5 months from his final octreotide dose. A 7-week post-treatment MRI brain demonstrated stable disease with 11.5% reduction per RANO-Meningioma and a 2.6% reduction per RECIST 1.1 criteria. Combined PRRT and octreotide represents a promising therapeutic strategy for patients with refractory meningioma.

## 1 Introduction

The majority of meningiomas exhibit indolent behavior, but a subset of these tumors demonstrates aggressive behavior (1). Surgical resection remains the first-line therapy for meningiomas, with adjuvant radiation therapy (RT) improving progression-free survival (PFS) for these tumors (2). However, toxicity risks have prompted further investigations into individualized approaches to therapy (3, 4). Evidence is lacking for systemic therapies with no established Phase 3 evidence and benefit is limited (**Table 1**) (20). Agents, including bevacizumab and sunitinib demonstrate modest disease control, and are associated with intratumoral hemorrhage (6, 16, 21, 22). For these reasons, targeted approaches are under active investigation, though most data are derived from small, early-phase or retrospective studies. Moreover, genomic driver mutations are variable across meningiomas and may be absent entirely.

**Table 1:** Systemic therapies for refractory meningioma.

Systemic therapy	Study title	Patients (n)	Outcome	
Abemaciclib 200mg BID daily	Alliance A071401: Phase II trial of abemaciclib in patients with grade 2/3 meningiomas harboring somatic NF2 or CDK pathway alterations (5)	36 (28 Grade 2, 8 Grade 3)	PFS-6	54%
Bevacizumab (10 mg/kg IV every 2 weeks for 6 months, then 15 mg/kg IV every 3 weeks)	A multi-institutional phase II trial of bevacizumab for recurrent and refractory meningioma (6)	42 (10 Grade 1, 20 Grade 2, 12 Grade 3)	PFS-6	87% Grade 1, 77% Grade 2, 46% Grade 3
Gefitinib (500–1000 mg PO continuous daily) erlotinib (150 mg PO continuous daily)	Phase II trials of erlotinib or gefitinib in patients with recurrent meningioma (7)	25 (16 gefitinib, 9 erlotinib)	PFS-6	25% benign, 29% Grade 2/3, 25% gefitinib, 33% erlotinib
GSK2256098 (750 mg PO BID)	Alliance A071401: phase II trial of focal adhesion kinase inhibition in meningiomas with somatic *NF2* mutations (8)	36 (12 Grade 1, 24 Grade 2/3)	PFS-6	83% Grade 1, 33% WHO Grade 2/3
Hydroxyurea (500 mg PO BID) Imatinib (400–800 mg PO daily)	Phase II study of Gleevec® plus hydroxyurea (HU) in adults with progressive or recurrent meningioma (9)	21	PFS-6	61.9% (87.5% Grade 1, 46.2% Grade 2/3)
Imatinib (600 mg/day for the initial 4-week cycle and then 800 mg/day for subsequent cycles)	Phase II study of imatinib mesylate for recurrent meningiomas (10)	23 13 benign (WHO grade 1), 5 atypical (WHO grade 2), 5 malignant (WHO grade 3))	PFS-6	29.4% (45% for benign, 0% Grade 2/3)
Mifepristone (200 mg PO daily)	Double-BLIND PHASE III randomized trial of the antiprogestin agent mifepristone in the treatment of unresectable Meningioma: SWOG S9005 (11)	164 (80 mifepristone, 84 placebo)	PFS-24	30% mifepristone, 33% placebo
Nivolumab (240mg IV q2weeks)	Phase II trial: activity of PD-1 blockade with nivolumab among patients with recurrent atypical/anaplastic meningioma (12)	25	PFS-6	42.40%
Octreotide (30 mg LAR IM monthly) and Everolimus (10 mg PO daily)	Combination of everolimus and octreotide LAR in aggressive recurrent meningiomas (13)	20	PFS-6	55%
Pasireotide (60 mg LAR IM monthly)	Phase II study of monthly pasireotide LAR (SOM230C) for recurrent or progressive meningioma (14)	34 (16 benign,18 Grade 2/3)	PFS-6	50% benign, 17% Grade 2/3
PTK787/ZK 22585 (500 mg PO BID)	A phase II trial of PTK787/ZK 222584 in recurrent or progressive radiation and surgery refractory meningiomas (15)	22 (14 Grade 2, 8 Grade 3)	PFS-6	64.3% Grade 2, 37.5% Grade 3
Sunitinib 50 mg PO daily	Phase II trial of sunitinib for recurrent and progressive atypical and anaplastic meningioma (16)	36	PFS-6	42%
Temozolomide (75 mg/m^2^ for 42 consecutive days every 10 weeks)	Temozolomide for treatment-resistant recurrent meningioma (17)	16	PFS-6	0%
Trabectedin (1.5 mg/m^2^ every 3weeks)	Trabectedin for recurrent WHO grade 2 or 3 meningioma (18)	90 (61 trabectedin, 29 local standard of care)	PFS-6	2.4 months trabectedin, 4.2 months local standard of care
Vistusertib (125 mg PO BID for 2 days per week)	Prospective phase II trial of the dual mTORC1/2 inhibitor vistusertib for progressive or symptomatic meningiomas in persons with neurofibromatosis 2 (19)	18	Volume decrease > 20%	6% PR, 94% SD

BID, twice daily; IV, intravenous; NF2, neurofibromatosis; PD-1, programmed cell death 1; PFS, progression-free survival; PR, partial response; SD, stable disease.

Accordingly, somatostatin receptor 2 (SSTR) analogs, including octreotide, are an attractive therapeutic given the ubiquitous expression of SSTR2 in nearly all meningiomas (23, 24). Octreotide exerts anti-proliferative effects, but has shown inconsistent clinical efficacy with a generally modest PFS-6 of 44% and frequent disease progression (14, 25–29). Resistance mechanisms, including intracellular downstream activation of the PIK3CA pathway may limit efficacy (13, 25). Combination octreotide with everolimus, a mammalian target of rapamycin inhibitor, has demonstrated an incremental benefit of PFS-6 to 55% (13).

As such, novel therapies, including SSTR-targeted peptide receptor radionuclide therapy (PRRT), with mechanisms independent of intracellular signaling have emerged (30). ^177^Lu-DOTATATE is one such PRRT with a favorable toxicity profile (30–32). β particles induce cytotoxicity by generating reactive oxygen species, thereby leading to single- and double-strand DNA breaks (31, 33, 34). Interim results from a Phase 2 study investigating PRRT monotherapy showed a PFS-6 rate of 50% in 14 refractory meningioma patients with acceptable toxicity and stable disease (SD) as the best response (30), thereby substantiating historical results of other single-agent PRRTs for the treatment of meningiomas (**Table 2**).

**Table 2:** Single-agent PRRT for the treatment of refractory meningioma.

PRRT	Design	Study title	Patients (n)	Outcome	
90Y-DOTATOC or ^177^Lu-DOTATATE	Prospective	Long-Term Results of a Phase II Clinical Trial: Somatostatin Receptor–Targeted Radiopeptide Therapy with 90Y-DOTATOC and ^177^Lu-DOTATATE in Progressive Meningioma (35)	34 (8 Grade 1, 12 Grade 2, 14 Grade 3)	SD	68% (23/34)
90Y-DOTATOC or ^177^Lu-DOTATATE	Retrospective	Somatostatin receptor-targeted radionuclide therapy for progressive meningioma: benefit linked to 68Ga-DOTATATE/-TOC uptake (36)	20 (5 Grade 1, 7 Grade 2, 8 Grade 3)	PFS-6	100% Grade 1, 57% Grade 2, 0% Grade 3
90Y-DOTATOC or ^177^Lu-DOTATATE	Retrospective	Peptide Receptor Radionuclide Therapy in Advanced Refractory Meningiomas: Efficacy and Toxicity in a Long Follow-up (37)	42: 11 Grade 1 24 Grade 2 4 Grade 3 3 unknown	mPFS	16 m (27.2m Grade 1, 11.5m Grade 2/3
^177^Lu-DOTATATE	Prospective	Evaluation of the SSTR2-targeted Radiopharmaceutical ^177^Lu-DOTATATE and SSTR2-specific ^68^Ga-DOTATATE PET as Imaging Biomarker in Patients with Intracranial Meningioma (30)	14 (2 Grade 1, 11 Grade 2, 1 Grade 3)	PFS-6	50%
^177^Lu-DOTATATE	Prospective	A Prospective, Phase II Study of ^177^Lu-DOTATATE in Patients with Surgery- and Radiation-Refractory Meningioma: Results of the WHO Grade II/III Cohort (38)	20 (19 Grade 2, 1 Grade 3)	PFS-6	77.8%

In NET, combining octreotide with ^177^Lu-DOTATATE significantly improves PFS compared to single-agent octreotide (not reached vs 8.5 months) with an 11.7 month trend toward improvement in median overall survival (39), supporting a synergistic strategy to overcome resistance. This lack of statistical significance was attributed to the negative impact of the crossover from the single-agent octreotide arm to the combination arm (39). However, the results were considered clinically meaningful (39). These results served as the basis for FDA approval of combined ^177^Lu-DOTATATE and octreotide for advanced midgut SSTR-expressing NETs (40). These findings were subsequently validated in the NETTER-2 trial, which demonstrated significantly prolonged PFS in patients with gastroenteropancreatic (GEP) NETs treated with combined PRRT and octreotide compared with octreotide monotherapy (22.8 months vs 8.5 months) (41). While this combination therapy has not been formally evaluated in meningiomas, it represents a rational and promising approach for refractory meningiomas. Furthermore, combination therapy compared to single-agent therapy to overcome resistance mechanisms has shown success in the treatment of other primary CNS tumors, including glioblastoma and papillary craniopharyngioma (42, 43).

We present the first reported case of a heavily pre-treated *NF2-*mutant refractory meningioma patient who was managed with salvage combined theranostic PRRT and octreotide. A 7-week post-treatment brain magnetic resonance imaging (MRI) demonstrated SD with tumor regression (an 11.5% reduction as assessed by RANO-Meningioma and a 2.6% reduction as assessed by RECIST 1.1 criteria).

## 2 Case description

**Figure 1:**
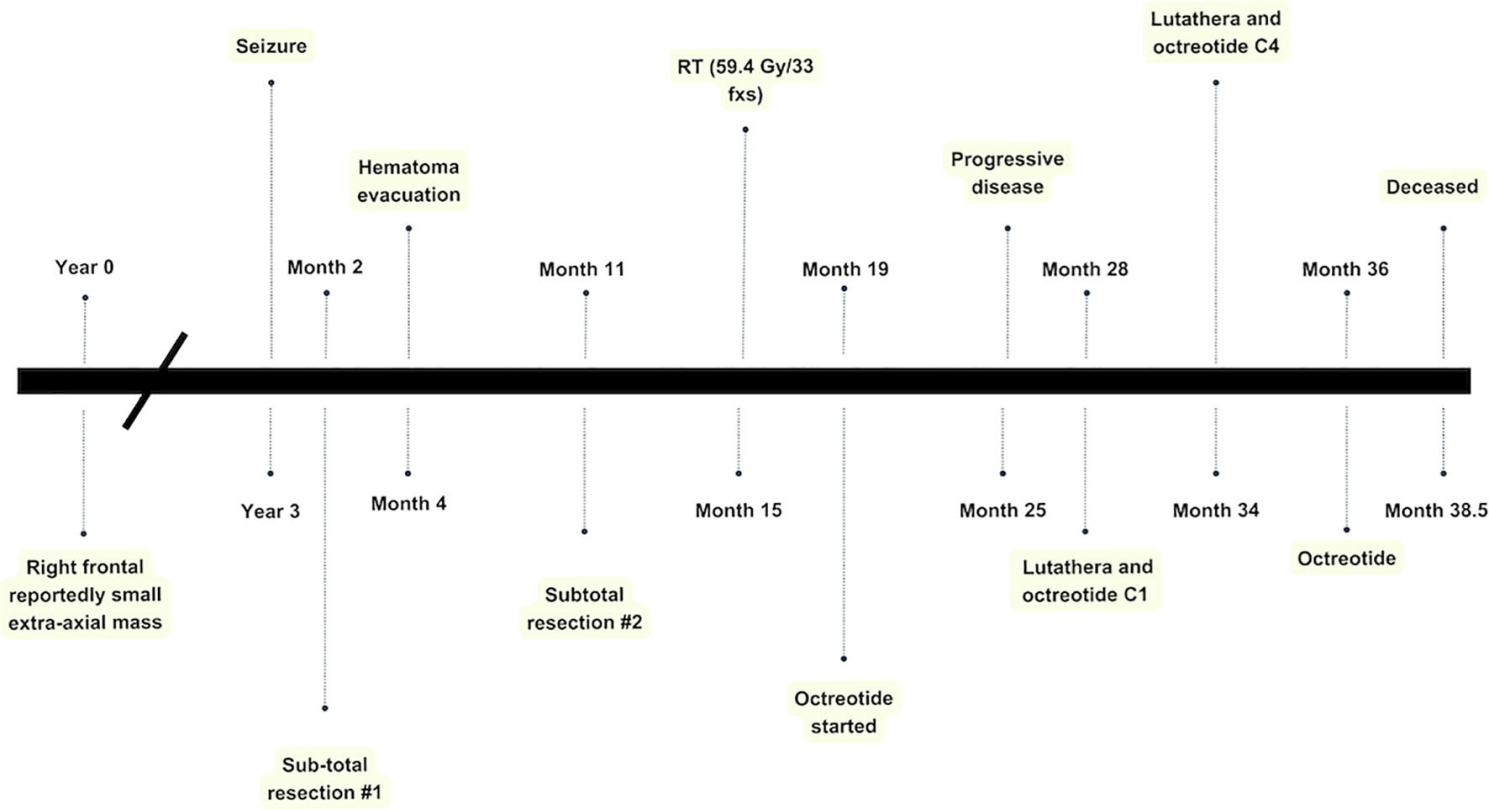
Patient disease course.

**Figure 2:**
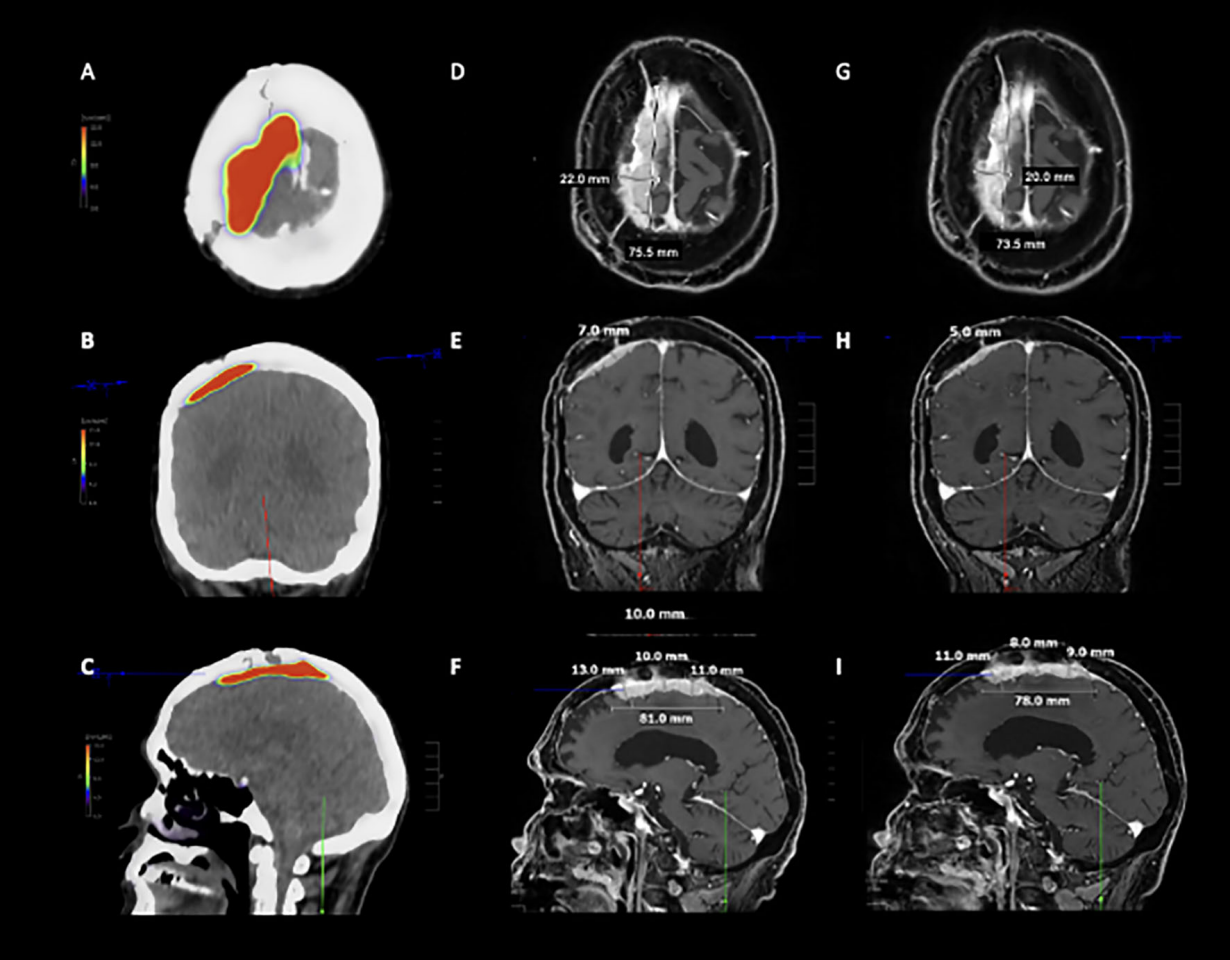
Pre-treatment 68Ga-DOTATATE PET **(A-C)**, brain MRI **(D-F)**, and 7-week post-treatment brain MRI **(G-I)** following 4 cycles of PRRT and octreotide.

A 66-year-old male presented to an outside facility following a fall (**Figure 1**). A brain MRI revealed a right frontal extra-axial homogeneously enhancing mass. He was recommended for observation, but was subsequently lost to follow-up. Three years later, he experienced a generalized tonic-clonic seizure. A repeat brain MRI scan demonstrated a 3.4 cm right frontal enhancing extra-axial mass, and observation was again recommended. Two months later, the patient presented with a left focal motor seizure and underwent a subtotal resection. Histopathological analysis demonstrated a Grade 1 meningioma. Given the benign findings, active surveillance was recommended. Two months later, the patient developed a right frontal epidural hematoma requiring evacuation. Six months later, tumor progression necessitated a re-resection. Histopathology of the recurrent tumor revealed malignant transformation to a Grade 2 atypical meningioma, characterized by brain invasion and 8 mitoses/10 high power fields. The Ki-67 index was 3-7% focally. Immunohistochemistry was positive for SSTR2 and negative for SOX10 and STAT6. Molecular analysis identified a pathogenic variant in *NF2* (NM_000268.3: c.70delG, p.V24fs), intact *CDKN2A/B*, and indeterminate *TERT* promoter mutation status. Tumor mutation burden was low (5 mutations/megabase), and PD-L1 expression was 0%. Chromosomal microarray analysis revealed multiple genomic alterations, including loss of heterozygosity (LOH) of 1p36.33-p11.2 (including *ARID1A*), gain of 1q21.1-q44, loss of 6q12-q27, loss of 9p24.3-p11.2 (including *CDKN2A/B*), LOH of chromosome 10 (including *PTEN* and *SUFU*), and LOH of chromosome 22 (including *NF2*). One month following adjuvant radiation therapy (59.4 Gy in 33 fractions), the patient was hospitalized for breakthrough seizures, weakness, gait instability, and urinary incontinence. A subsequent brain MRI showed a stable tumor with mild surrounding edema. A DOTATATE positron emission tomography (PET) scan demonstrated mildly avid tumor anteriorly with a maximum standardized uptake value (SUV_max_) of 3.6 and moderately avid tumor posteriorly with an SUV_max_ of 8.2 (**Figure 2**). An electroencephalogram demonstrated right hemispheric potential epileptogenic dysfunction consistent with a focal onset seizure disorder. Due to dysphagia, oral medications, including everolimus and sunitinib, were contraindicated. Sunitinib and bevacizumab were contraindicated due to his prior intracerebral hemorrhage and current anti-coagulation use. Consequently, dual somatostatin analog therapy with PRRT and octreotide was selected as salvage therapy. Due to delays in obtaining PRRT, intramuscular octreotide monotherapy was initiated with an initial dose of 30mg, followed by 40mg monthly for 7 months. A surveillance brain MRI demonstrated a 75.5mm x 22.0mm tumor. He then underwent four cycles of combined intravenous ^177^Lu-DOTATATE 7.4 GBq (200 mCi) and intramuscular long-acting octreotide 40 mg every 8 weeks. Treatment was well-tolerated with no treatment-related adverse events, other than Grade 2 lymphopenia that resolved spontaneously and Grade 1 nausea, which was managed with as needed ondansetron. Response assessment on 7-week post-treatment brain MRI demonstrated stable disease with a lesion measuring 73.5 × 20.0mm. This corresponded to an 11.5% reduction in tumor size per RANO-Meningioma and a 2.6% reduction per RECIST 1.1 criteria. A 4-week post-treatment DOTATATE PET/computed tomography scan showed increased, intense avidity, with an SUV_max_ of 31.1 (anteriorly) and 23.6 (posteriorly), with plans to obtain a 3-month post-treatment DOTATATE PET for suspected pseudoprogression. The patient continued on octreotide monotherapy for only 1 cycle due to his unfortunate and unexpected passing from septic shock that was unrelated to his tumor or tumor-directed therapy. His death occurred 2.5 months from his last octreotide injection. Ethical guidelines set out by the Declaration of Helsinki were followed in the preparation of this report, and the patient provided written consent.

## 3 Discussion

Meningiomas are provisionally diagnosed with MRI (1). However, MRI is suboptimal compared to SSTR2-targeted PET in distinguishing viable tumor from post-surgical or radiation treatment change (44). Compared with MRI, SSTR2-targeted PET demonstrates superior sensitivity and specificity, particularly in clinical scenarios of bone invasion, en plaque growth, skull base or parasagittal involvement (45–49). Recent consensus guidelines from leading consortia recommend SSTR2-targeted PET for the diagnosis, surgical planning, radiotherapy (RT) target delineation, and post-treatment surveillance of meningiomas (45, 50–52). According, SSTR2-targeted PET was useful in our patient to delineate viable tumor from radiation-related changes.

A subset of meningiomas do not follow the biologic behavior of their assigned grade. Our patient’s tumor did not follow the expected course of a benign Grade 1 meningioma. His tumor recurred and transformed within 6 months of initial diagnosis. However, the mitotic count was on the lower limit of the broad range for Grade 2 meningiomas (4–19 mitoses/10 HPF). Moreover, there were no Grade 2 histologic features of sheet-like growth, hypercellularity, small cell change, prominent nucleoli, or spontaneous necrosis (53). Although brain invasion was noted in his tumor and is a criterion that is independently sufficient for a Grade 2 designation, the prognostic relevance of brain invasion remains under debate. The CNS Tumor Taxonomy-Not Official WHO consortium revised the prior brain invasion diagnostic criterion with the introduction of a brain-invasive but otherwise benign meningioma (53, 54).

Though histopathological examination is currently the foundational approach for diagnosing meningiomas, recent efforts have led to a surge in molecular and epigenetic investigations, uncovering novel diagnostic biomarkers and prognostic genomic alterations (55–59). Further molecular analyses of our patient’s tumor provided complementary data to address the shortcomings of the existing grading criteria. His tumor harbored *NF2* loss-of-function mutations. Though *CDKN2A/B* was intact, further analysis with chromosomal array analysis revealed loss of 9p24.3p11.2, including *CDKN2A/B*, which mandates a Grade 3 assignment. Furthermore, an abundance and accumulation of somatic copy number alterations, including 1p, 6q, 10, 11p, 13q, 18, and Y loss; loss of heterozygosity of 14 and 22; and gain of 21 were uncovered and may explain the aggressive nature of his histologically benign tumor (54, 60). This genomic instability is most commonly observed in *NF2*-mutant meningiomas and is associated with progressive tumors (54). Furthermore, proliferative meningiomas harbor a high burden of copy number variants and losses of chromosomes 1p, 10, 14, 18 and 22q (3), all of which were uncovered in our patient’s tumor. Lastly, emerging evidence suggests that wildtype *NF2* meningioma subtypes derive benefit from RT (3, 4) while *NF2* loss-of-function mutations confer radio-resistance (3, 61, 62), which may provide reasoning for the progression in our patient’s *NF2*-mutated tumor following RT.

An optimal threshold for SUV_max_ of 2.3 or a ratio in comparison to the superior sagittal sinus SUV(R_sss_) of 3.0 have been defined to identify viable tumor (47, 49, 63). Other studies suggest that a 3-fold increase in SUV_max_ compared to the SSS is likely to represent tumor, while those with a less than a 3-fold increase in SUV_max_ suggest post-operative change (64). The pre-treatment DOTATATE PET was avid and exceeded both thresholds in his posterior tumor, supporting the use of somatostatin analogs. The multi-SSTR-targeted approach with octreotide and PRRT proven in the landmark NETTER-1 and NETTER 2 studies for SSTR2-positive GEP NETs (39, 41) was selected for our patient due to contraindications to and the limited efficacy of existing therapies. His 7-week post-treatment brain MRI showed promising results with tumor regression.

Though pretreatment SSTR PET carries demonstratable utility in detecting patients suitable for PRRT (65), the role of SSTR PET as a predictive biomarker for treatment response has not been established (65). His 4-week post-treatment SSTR PET may have been compromised by pseudoprogression as a result of SSTR2 upregulation (63, 65–69). The increase in avidity and accumulation does not confirm an SSTR2 upregulation nor treatment failure (63, 65–67). Most studies obtain a 2–6 month posttreatment SSTR PET for response assessment (30, 52, 65). The optimal timing for post-PRRT imaging remains under debate and there are no established guidelines for the timing of post-PRRT treatment to gauge the response to therapy (65, 67).

Combination PRRT and octreotide was tolerated well in our patient with no treatment-related adverse events greater than Grade 1, substantiating the previously reported favorable toxicity profile (30). This is the first reported case of a refractory heavily pre-treated meningioma treated with salvage combination PRRT and octreotide.

The significance of this study is highlighted by the lack of effective therapies for meningiomas, wherein systemic therapies are reserved for refractory cases. This unmet need remains a formidable gap in the existing treatment paradigm, despite an abundance of wide-ranging investigated systemic therapies. This study is innovative and explores unchartered areas of scientific knowledge by investigating the use of two concurrent SSTR-targeted therapies for aggressive meningiomas, with each therapy offering a distinct mechanism for cytotoxicity. This study builds upon the forward movement towards the adoption of PRRT in the management of meningiomas and may broaden the therapeutic options for refractory meningiomas. SSTR2 represents a universal target that is collectively found in nearly all meningiomas and is an integral component in the generalizability and scalability for treatment practice-changing initiatives. This multi-SSTR2 combination targeted therapy diverges from other targeted approaches for meningiomas, which are reliant on less commonly occurring driver mutations in select patients. In that setting, shifting to real-world adoption may be challenging. This study also explores the real-world use and the potential value of chromosomal analysis for meningiomas.

We acknowledge limitations in this study. First, an isolated case is not generalizable to all patients with refractory meningiomas. Second, this study is limited by the short-term follow-up due to the unexpected and unfortunate death of our patient from unrelated causes. Though PFS-6 is the established benchmark for assessing meningioma response to treatment, our patient’s tumor showed a reduction in tumor size on his immediate post-treatment MRI.

Future investigations will reveal whether or not PRRT monotherapy improves PFS-6 greater than 50% or SD as best response (30). Preliminary evidence from a Phase 2 Study investigating the use of single agent PRRT in 26 refractory Grade 2/3 meningiomas reported a PFS-6 of 77.8% (38). Prospective randomized multi-center Phase II studies investigating the use of single-agent PRRT for refractory meningiomas are currently underway in the European Organization for Research and Treatment of Cancer LUMEN-1 (NCT06326190) and Radiation Therapy Oncology Group MOMENTUM-1 (NCT06955169) trials (70).

## 4 Conclusion

Beyond local therapies, no standard of care therapy exists for refractory meningiomas. Despite the ubiquitous expression of SSTR2 in meningiomas, single-agent octreotide and single-agent PRRT have yielded limited benefit. The FDA-approved dual SSTR2-targeted therapeutic strategy combining octreotide with PRRT has demonstrated clinical benefit in SSTR2-positive neuroendocrine tumors, as established in the NETTER-1 and NETTER-2 trials. We present the first reported case of a refractory meningioma treated with combined PRRT and octreotide demonstrating promising results.

## Data Availability

The original contributions presented in the study are included in the article, further inquiries can be directed to carleny@hs.uci.edu.
